# Assessing the environmental and climatic influences on the incidence of severe typhoid in Kampala, Uganda

**DOI:** 10.1371/journal.pgph.0004214

**Published:** 2025-04-17

**Authors:** John Bosco Kalule, Nakintu Zalwango Valeria, Majalija Samuel

**Affiliations:** Department of Biotechnical and Diagnostic Sciences (BDS), College of Veterinary Medicine Animal Resources and Biosecurity (CoVAB), Makerere University, Kampala, Uganda; Child Health Research Foundation, BANGLADESH

## Abstract

Typhoid is a water and foodborne febrile illness which often mimics malaria in endemic African nations. This study deployed a point-of-use water testing approach to assess the public health risk associated with the consumption of water from spring wells (open or closed wells) and boreholes in off-grid areas in Kampala, and then assessed the correlation between incidence of in-patient typhoid cases at the local health facilities, and monthly rainfall amounts in Kampala.We retrieved 10-year archived data on monthly incidence of severe typhoid in-patient cases and corresponding data on monthly rainfall amounts and evaluated the interrelation between monthly rainfall and the incidence of inpatient department cases using regression and time-series analysis. The Portable Microbiology Laboratory was used to determine the level of disease risk associated with currently used underground water sources in Kampala. There was positive correlation between monthly rainfall amounts and incidence of severe typhoid cases in Kampala with a strong seasonal component with consistent annual peaks. The surface water sources in Kampala pose moderate to severe disease risk to the user communities and should be monitored and tested for microbial quality to ensure public health safety. Typhoid incidence in Kampala is weather-sensitive and predictable. Environmental modifications and vaccination could prevent the strong annual peaks of severe typhoid.

**Highlights**

- There was positive correlation between monthly rainfall amounts and the incidence of severe typhoid cases in Kampala.
- Underground water sources in Kampala district in Uganda pose a moderate to very high disease risk to user-communities.
- Typhoid incidence in Kampala had a strong seasonal component with consistent annual peaks.

## 1 Introduction

Salmonella Typhi is responsible for typhoid fever in approximately 10.9 million people annually and is primarily spread through the consumption of contaminated water [1]. Studies suggest that in malaria-endemic regions, children presenting with fever, but who do not have malaria, often go without proper diagnosis and treatment, resulting in high mortality rates [2].

In Uganda, most rural and peri-urban communities rely on spring wells, boreholes, and roof top water for domestic use; these are not safely managed by way of chlorination and laboratory testing for quality. Water from these sources was found to hold *E. coli* counts higher than is recommended by the World Health Organization (WHO) and the Uganda national water standards [3].

Kampala district frequently experiences occasional typhoid fever outbreaks, presumably due to limited access to piped chlorinated water, with only a few households benefiting from it [4].

Typhoid and paratyphoid fever may exhibit a seasonal pattern, albeit which is not well defined. The environmental factors driving these seasonal variations are not fully understood, but growing evidence indicates that rainfall and temperature could play a significant role [5].Typhoid outbreaks often occur during the summer months, with higher temperatures accelerating the growth and transmission of S. Typhi through contaminated food. Relatedly, regions with warmer climates tend to experience higher incidences of typhoid compared to those at higher latitudes [6]. Crucially, the underlying mechanisms that drive the seasonality of enteric fever are likely dependent on the local context and should be taken into account when designing control efforts. Kampala is endemic for typhoid but occasionally suffers outbreaks. The driving environmental factors behind these outbreaks are not known. In some instances, typhoid and paratyphoid fever patients present with less severe illnesses and are managed from home; but some of them present with severe typhoid and are managed as inpatients [7].

We hypothesize that the rise in precipitation during the rains increases the probability of typhoid pathogens congregating in shallow waters and contaminating the water table leading to long cycle transmission of S. Typhi following incubation in the surface water such that the outbreaks happen at the beginning of the dry spell. Kampala floods could create vulnerability to fecal‐oral transmission of typhoid due to obstructed urban drains and the release of untreated sewage into the stormwater drains.

The 100 ml membrane filtration test is widely used by the national water quality monitoring programs in Africa for bacteriological quality assessment usually following chlorination where no coliforms or *E. coli* are expected [8]. The Portable Microbiology Laboratory (PML) is a WHO-approved water testing algorithm which can be used at the community level with no requirement for advanced laboratory equipment and skilled labor. It includes the Colilert® 10 ml presence/absence test and the quantitative 1.0 ml *E. coli*/Coliform Count Petrifilm^TM^ (3M, St. Paul, MN). These two tests can easily be conducted in the field following brief training, providing overnight results through body incubation and categorizing the source based on WHO’s low to very high-risk categories without the need for filtering equipment [9].

In this study we used seasonal decomposition for insights into the trend, seasonal patterns, and residuals of the time series, ARIMA modeling to investigate how past values of rainfall and in-patient department (IPD) cases predict future values, and then cross-correlation to reveal how past rainfall would impact future IPD cases. Subsequently, since rainfall directly affects quality of underground water reservoirs, we tested them for the level of disease risk they pose to the user community using the PML.

## 2 Methods

### 2.1 Ethics statement

This study was approved by the Research and Ethics Committee of the School of Biotechnical, Biomedical, and Laboratory Sciences at Makerere University, Kampala (HREC 376/024). The research utilized archived data and did not involve direct human participant contact. All patient-related data were fully anonymized prior to analysis to ensure confidentiality and privacy.

### 2.2 Study setting

Kampala district is situated at an elevation of 1,211 meters above sea level, with a population of approximately 3.5 - 4 million people and 1.5 million households. It has a tropical rainforest climate, with an average annual temperature of 24°C and receives about 1,260 millimeters of precipitation annually. All the health centers in Kampala district report the number of confirmed typhoid cases detected in their facilities to the Kampala Capital City Authority health division. Data on the monthly rainfall amounts was collected by the Entebbe Meteorological Station.

### 2.3 Water sample collection

**Fig 1:**
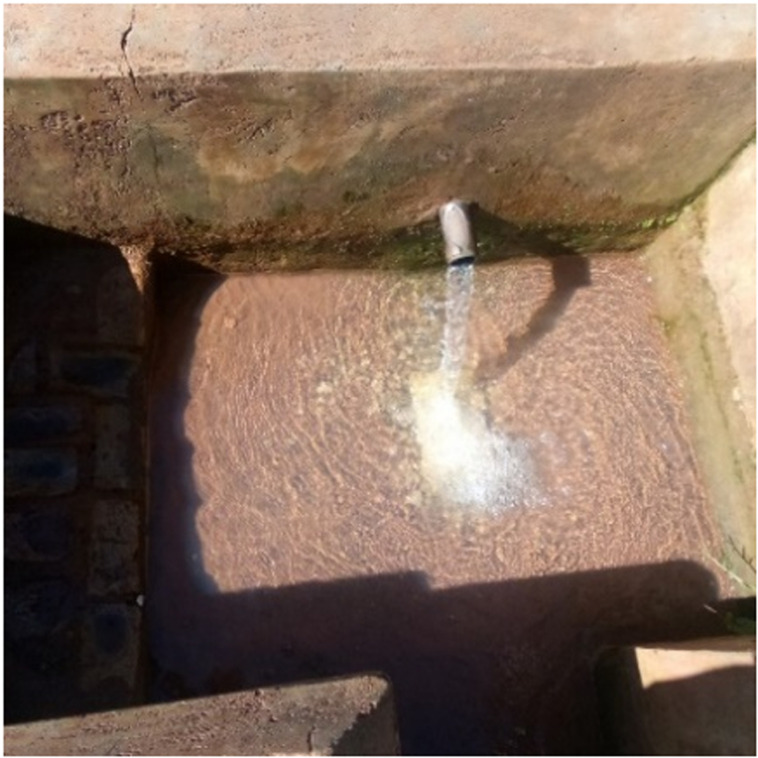
Closed spring well in Kampala.

The samples were collected from unbuilt spring wells (the top of these wells is not covered) using the depth-integrated-grab sampling technique at 5 cm below the water surface. The mid-stream sample collection method was used for the collection of water samples from built spring wells (spring wells with a covered top). For each well, the distance from the nearest was measured using a 100-meter measuring tape and recorded. (**Fig 1**)

**Fig 2:**
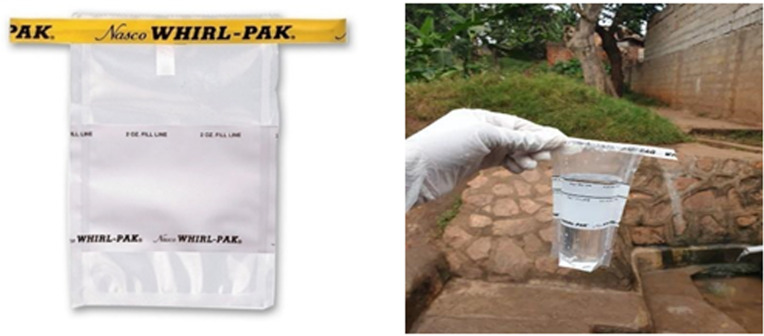
Collection of water sample from a built water source into a zip-locked 100ml whirl Pak using sterile technique.

Water samples were collected in sterile 100 ml whirl Pak containers (**Fig 2**). Information on the location of the water source, the estimated distance from the nearest homestead, and the date and time of sample collection were recorded on the container. The collected samples (100 ml) were then transported at 4°C in a temperature monitored box and processed on-site within one hour of collection.

### 2.4 Retrieval of data on the monthly rainfall amounts and monthly number of in-patient department typhoid cases in Kampala

To assess correlation between the monthly rainfall amounts and the incidence of typhoid, data on the monthly rainfall amounts and in-patient typhoid cases were retrieved from the Entebbe Meteorological Station (EMS) and Kampala Capital City Authority (KCCA) public health division respectively for the period January 2008 to August 2019 and cleaned in Microsoft Excel. (Microsoft Corporation, 2023). Only laboratory-confirmed in-patient typhoid cases are recorded by the Kampala Capital City Authority (KCCA) Public Health Division. Diagnostic protocols for typhoid in this setting include blood culture for pathogen isolation, followed by confirmatory identification using the VITEK II system (bioMérieux, France) [4].

### 2.5 Point-of-use testing using the PML kit and characterization of *E. coli* colonies

To determine which water sources, pose a disease risk to the user communities, we conducted examinations on subterranean water sources in Kampala district in Uganda from July to December 2021. A total of 38 underground water sources including spring wells and boreholes were tested using the PML according to the WHO disease risk guidelines.

**Fig 3:**
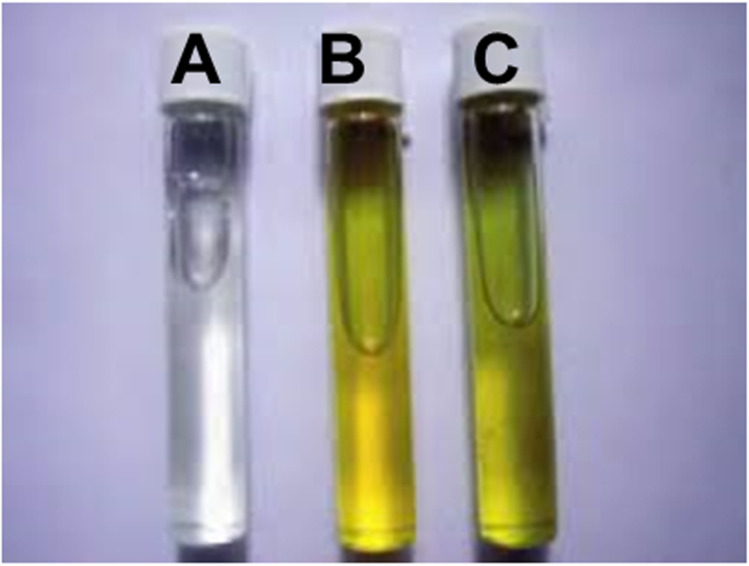
Interpretation for the Colilert test after 12-18h incubation at body temperature. A clear tube showed no coliforms present **(A)**, a yellow tube with no fluorescence under long wave UV light indicates coliform bacteria other than *E. coli* are present **(B)**. A yellow tube with blue fluorescence under long wave UV light showed that *E. coli* was present **(C)**.

Field-based testing was carried out by the community members with the supervision of a microbiologist as described below. The testing included both quantitative and qualitative (presence/absence) approaches. Of the 100 ml of water sample collected, 10 ml of sample were aspirated using a 1ml pipette into the Colilert® 10ml presence/absence test tube (IDEXX, Westbrook, ME). The tube was then inverted four times to form a homogenized mixture before incubation at body temperature (35–37°C) for 12–18 h in a black opaque purse-string bag. Interpretation of the results of this test was based on the occurrence or absence of a color change to yellow and/ or florescence when flashed with UV light from a battery-operated handheld lamp. (**Fig 3**)

To carry out the *E. coli*/ Coliform Count Petrifilm™ (3M, St Paul, MN) test, a sterile 1 ml pipette was used to dispense 1ml of sample into the center of the 5 cm nutrient circle on the Petrifilm ensuring that folding back the plastic layer on the nutrient surface doesn’t trap air bubbles. Subsequently, uniform spreading of the inoculum was done using a plastic spreader (3M, St Paul, MN). The petrifilm was then sandwiched between two paper carboard quadrants and fastened with an elastic rubber band before incubation at body temperature [9].

The results were then interpreted according to the World Health Organization’s guidelines on drinking water quality. (**Table 1**)

**Table 1:** Disease Risk Level as per the WHO guidelines for Drinking water quality.

*E. coli* in sample	Colilert MUG+	*E. coli* colonies on Petrifilm	Disease Risk level
<1/10ml	–	0	Low
1–10/10ml	+	0	Moderate
1–10/ml	+	1-10	High
>10/ml	+	>10	Very high

*The Colilert and Petrifilm tests are specific for *E. coli* because they contain a substrate for the betaglucoronidase enzyme that is produced by *E coli* but not by the environmental coliform bacteria.

### 2.6 Quality control

**Fig 4:**
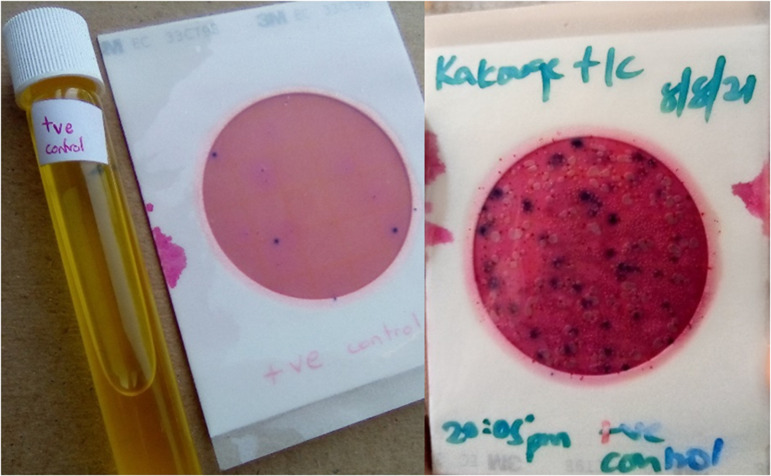
Positive controls used for the Colilert presence/absence test and the E. coli/ Coliform count Petrifilm test.

Before use, the Colilert tubes and the *E. coli*/ Coliform Count Petrifilm™ plates (3M, St Paul, MN) were kept away from direct sunlight using a cardboard box. For each batch of test samples, a positive (**Fig 4**) and negative control were run along with the test sample batch. Sterile laboratory grade water was used as the negative control while an inhouse positive control of sterile laboratory grade water spiked with confirmed *E. coli* was used as the positive control. The controls and test samples were incubated under similar conditions using body temperature.

### 2.7 Data analysis

The mean *E. coli* counts per ml and the comparison of proportions was done using the open- source public health statistical software called OpenEpi [10]. The two proportion z test was used to compare proportions. To determine the impact of rainfall and seasons on the current and future IPD cases, we carried out regression and time series analysis using the R software.

## 3 Results

### 3.1 Typhoid cases and rainfall amounts in Kampala

**Fig 5:**
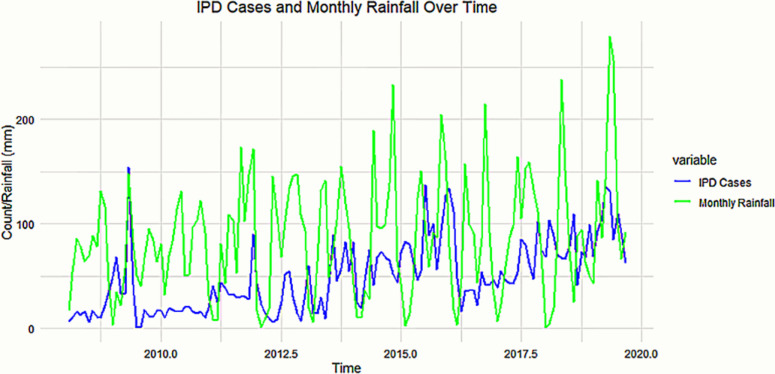
Time series of Inpatient Department cases with monthly rainfall amounts.

Preliminary data on the incidence of typhoid fever in Kampala, with an incidence rate of approximately 160 cases per 100,000 person-years, was obtained from the Health Division of the Kampala Capital City Authority (KCCA) [7]. The monthly incidence of typhoid inpatient cases fluctuated depending on the season. More cases were observed in the wet compared to the dry season. The prominent peak observed between 2014 and 2015 represents a typhoid outbreak. The data show that overall, the number of typhoid cases increases overtime probably with the changes in climate. Since 2014, there has been a general increase in the monthly occurrence of typhoid cases reported. (**Fig 5**)

### 3.2 Testing of surface water sources in Kampala district

A total of 38 water samples were collected from as many water sources. Of these, 42.1% (16/38) were collected from boreholes while 57.9% (22/38) were collected from wells. The mean *E. coli* count/ml for wells was generally higher than that for boreholes.

Of the 16 boreholes tested 15 were low disease risk while one was of moderate disease risk. Of the 22 wells, 40.9% (9/22) posed a very high disease risk to the user community. (**Table 2**).

**Table 2:** Numbers of boreholes and wells that were sampled in Nakasongola and Kawempe.

	Kampala		
	Boreholes	Wells	Total
Low	15	7	22
Moderate	1	3	4
High	–	3	3
Very high	–	9	9
Total	16	22	38

The proportion of wells (15/22) that posed a moderate to very high disease risk to the community was significantly higher (p-value= 0.0001347) than that for boreholes (1/16).

#### 3.2.1 Regression analysis.

**Fig 6:**
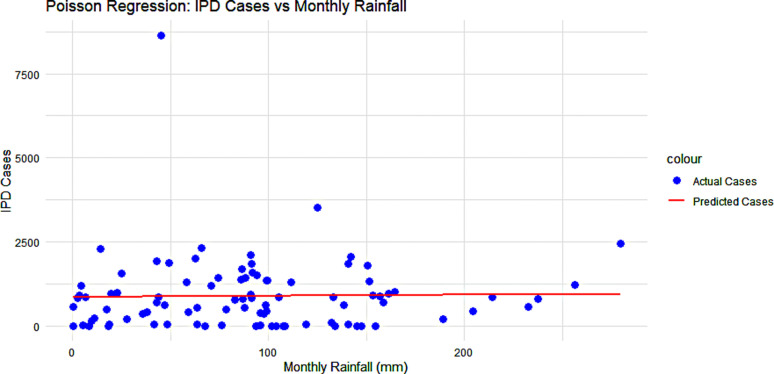
Poisson Regression showing Inpatient-Department cases vs monthly rainfall amounts.

The expected number of IPD cases when the monthly rainfall is 0 was approximately 872 cases. (Intercept = 6.773, p-value =<2e-16). The coefficient for monthly rainfall which represented the log change in the expected number of IPD cases for a one-unit (1mm) increase in rainfall, the expected number of IPD cases increases by 0.0375% (p-value = 2.11e-11). The extremely small p-value signifies that monthly rainfall is a significant predictor of the number of IPD cases and that it affects the number of typhoid cases. Even though the effect is small it’s not due to random chance. (**Fig 6**)

### 3.3 Time series analysis

#### 3.3.1 Decomposition of an additive time series.

The trends reveal that there was a major increase in typhoid cases around 2015, possibly due to an outbreak, followed by stabilization. The trend line isolates the long-term pattern in the data, smoothing out the short-term fluctuations. There was a strong upward trend starting around 2013, peaking around 2015, followed by a slight dip and stabilization afterward. This suggests a significant increase in typhoid cases over these years, possibly due to environmental or demographic factors. In the later years, the trend slowly rises again, indicating a steady increase in cases.

Regarding seasonality, the data shows a strong seasonal component, with consistent annual peaks, indicating that certain months or seasons are prone to more cases (likely linked to environmental factors like rainfall). The seasonal component captured periodic, repeating patterns that occur within each year. There is a clear annual pattern, showing higher cases at specific times each year. The peaks seem to occur around the same time every year, implying that typhoid cases have a strong seasonal effect, potentially tied to seasonal rainfall or temperature changes. This pattern is consistent year-to-year, confirming the seasonality in typhoid outbreaks.

**Fig 7:**
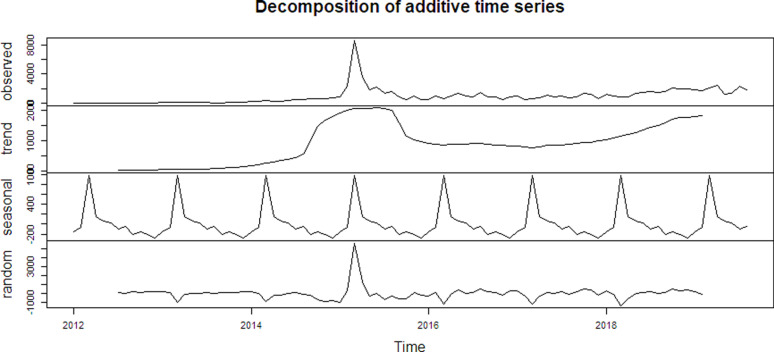
Decomposition of additive time series.

The residual or random component is relatively small except for the spike in 2015, suggesting that the trend and seasonal components explain most of the data’s variability. (**Fig 7**)

#### 3.3.2 Forecasts from ARIMA (Auto-Regressive Integrated Moving Average).

The ARIMA(0,1,2) model forecasts stability in future IPD cases, with no expected dramatic increases or decreases based on past data trends.

**Fig 8:**
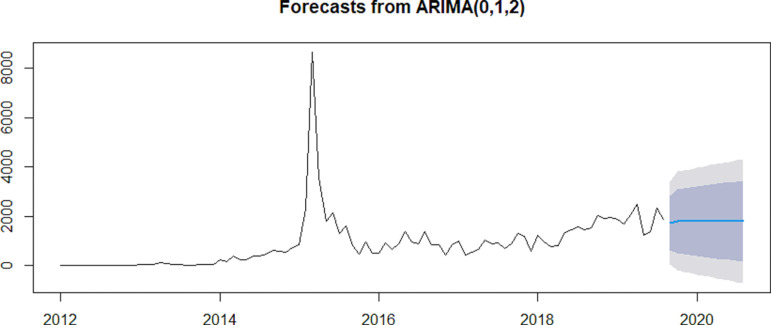
Forecasts from ARIMA for future In-patient Department cases.

The model doesn’t capture any strong seasonality or recurring patterns, meaning that it doesn’t anticipate the kind of large spike seen around 2015, which could indicate that the spike was an unusual event not expected to repeat. (**Fig 8**)

**Fig 9:**
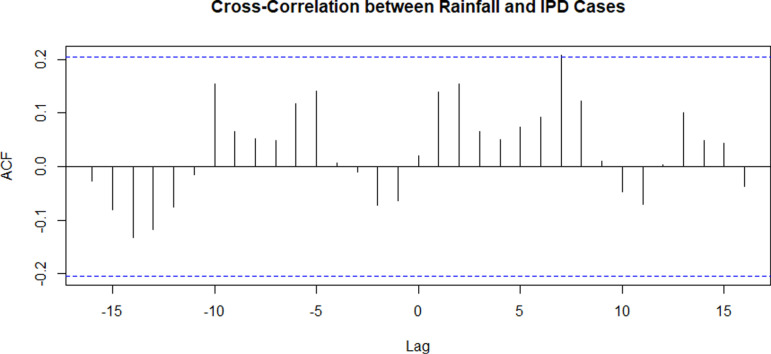
Cross-correlation between rainfall and In-patient Department cases.

At negative lags (-5 to -10), there was some significant positive correlations, suggesting that rainfall from 5 to 10 months earlier may be associated with an increase in future IPD cases. This could imply a delayed effect of rainfall on typhoid incidence. (**Fig 9**)

## 4 Discussion

The UN-Water Integrated Monitoring Initiative for SDG 6 (IMI-SDG6) encourages nations to monitor water- and sanitation-related issues by compiling country-level data and report on progress towards SDG 6. As per the UN-Water performance indicator 6.1.1, by 2030, each household should have access to a water source which is located on the premises, available when needed, and free of fecal and priority chemical contamination. The NWSC routinely monitors the quality of chlorinated piped water using the 100ml membrane filtration test [11] but its scope is limited to mainly treated water in the urban areas. Off grid zones in rural and peri-urban areas with no access to piped water resort to the use of underground water sources such as spring wells and boreholes; but these are not part of the testing scope of NWSC. The community in Kampala has frequently suffered typhoid outbreaks; but the exact environmental source of these outbreaks has never been identified [12]. Previous studies showed that underground water in both rural and urban areas in Uganda was not fit for human consumption and carried high loads of *E. coli* [13, 14] Another study that assessed the risk factors for underground water contamination found that there was a significant relationship between median level of contamination and rainfall, total sanitary score, and that population density was a confounding factor – findings that are in tandem with those of this study [15]. However, this study did not find a positive correlation between monthly rainfall amounts and incidence of typhoid cases. A related study reported an increased relative risk of typhoid was long-lasting after the rains [16]. In this study, poisson regression revealed that monthly rainfall was a strong predictor of severe typhoid in Kampala. We noted an upward trend of typhoid cases starting from 2013 culminating in an outbreak in 2015. The underlying factors to this increase are not clearly understood but could be linked to the drastic increase in the cost of piped water in around 2013 forcing the communities to resort to underground water use. Furthermore, the strong seasonal component with consistent annual peaks reveals that typhoid predictably peaks during the same months each year; probably due to the peaking of S. Typhi in environmental reservoirs driven by various environmental factors. Interestingly, cross-correlation between IPD cases and monthly rainfall amounts revealed that rains from 5 – 10 months back may have an influence on the future typhoid cases. This is expected because the annual peak of typhoid cases occurs at the start of the dry season. It has been hypothesized that the warmer temperatures facilitate the incubation of S. Typhi in the environmental reservoirs [17].

This study was conducted in an urban setting with a high level of anthropogenic activity, where the water table responds rapidly to short rains (within 48 hours) due to the permeable and shallow vadose zone. The vadose zone is thin as a result of anthropogenic activities and has a limited capacity to attenuate contaminants [18]. Consequently, some diarrheal disease outbreaks have been associated with drinking unprotected well water contaminated with feces; [19] while others could not be linked to a single source [20].

Since the levels of underground water microbial contamination varied with seasons [18], its routine surveillance is of paramount relevance. One of the limitations to routine surveillance of underground water quality using the 100ml membrane filtration technique, is the expense, skill, and infrastructure requirement. According to UN-Water data for 2020, overall, only 17% of Ugandans have access to safely managed drinking water services. Only 8% of the rural inhabitants and 43% of the urban dwellers have access to safely managed drinking water services. The small coverage of the NWSC may be due to the limitations of the scope of water laboratory services and the associated logistics. For Uganda to promptly achieve SDG 6.1, there is need to alter water testing strategy in such a manner that testing can be done cheaply by the concerned rural communities. Access to safe drinking water in homes, healthcare facilities, schools and workplaces effectively reduces water-borne disease and malnutrition, which are leading causes of death among children under five [21]. The most important public health determinant for communities without piped, chlorinated water is the status of their drinking water. The PML tests enable communities and public health agencies to easily determine the disease risk of water sources, and to develop strategies to make sure that all citizens have access to low disease sources.

The PML has already been used as a key part of a strategy to eliminate waterborne disease in Lower Nyakach, Kenya, by the community-based organization, Friends of the old. A random sample of 377 households revealed that 95% treat their water every time they collect. Microbiological verification found 96% of household safe water storage vessels were low disease risk compared to their very high disease risk source water [22].

Coupled with onsite chlorine dispensers, household distribution of liquid chlorine or chlorine tablets, the use of the PML could potentially aid the control of waterborne disease in remote rural areas. Since household surveys and censuses (from the National Statistical Office) remain the primary source of information on the different types of water facilities used by the population, it would be prudent to empower the household members to assess the disease risk associated with the water they consume.

### 4.1 Study limitations

This study did not incorporate direct water sample testing for Salmonella Typhi. This would have provided more direct evidence of environmental reservoirs and improved our understanding of the transmission pathways of typhoid.

Unfortunately, our study lacked direct flood risk data specific to Kampala for the period covered in our study and yet it is a potential predictor of typhoid outbreaks and is important for typhoid fever disease modeling and prediction.

## 5 Recommendations

Environmental modifications in addition to vaccination using the typhoid conjugate vaccines should be adopted to prevent the consistent annual peaks of severe typhoid in Kampala district, Uganda. Further research is needed to investigate the underlying factors of the 2015 typhoid outbreak.

Communities that are heavily dependent on underground water sources for drinking water need to alter their water testing strategy in such a manner that its decentralized to the user-communities. A point-of-use water-testing strategy in waterborne disease outbreak-prone villages will allow the communities to evaluate surface water sources and the impact of the different interventions on water safety such as boiling and chlorination. The use of the PML as an alternative to the 100ml membrane filtration test will enable rural communities to cost-effectively access safely managed drinking water.

Public health authorities should consider high rainfall as an early warning signal for potential typhoid outbreaks, enabling them to take proactive measures, such as increasing surveillance and ensuring adequate resources for diagnosis and treatment.

There is need for future studies to integrate flood risk data, which could help enhance predictive models and improve public health interventions.

## 6 Conclusions

There is positive correlation between monthly rainfall amounts and incidence of severe typhoid cases in Kampala with a strong seasonal component with consistent annual peaks. The surface water sources in Kampala pose moderate to severe disease risk to the user communities and should monitored and tested for microbial quality to ensure public health safety.

## 7 Open research

All the data generated in this research is included in this manuscript and we did not use any new data. The software that was used in the analysis of the descriptive data presented in this manuscript is the open- source public health statistical software called OpenEpi [10].
